# Multi-channel pattern VEPs with full and half field stimulation: methods of interpretation and diagnostic evaluation

**DOI:** 10.1007/s10633-025-10012-7

**Published:** 2025-03-25

**Authors:** Dorothy A. Thompson, Oliver R. Marmoy, Joanne Cowe, Siân E. Handley

**Affiliations:** 1 Great Ormond Street Hospital for Children, NHS Foundation Trust, London, UK; 2 UCL Great Ormond Street Institute of Child Health, University College London, 30 Guildford Street, London, UK; 3 University Hospitals of Leicester NHS Trust, Leicester, UK

**Keywords:** Multichannel VEP, Pattern VEP, Half field stimulation, Paradoxical lateralisation, Chiasm, Hemisphere, Hemifield

## Abstract

**Aim:**

To describe methods of evaluating multichannel full and half field pattern VEPs using the ISCEV VEP Standard montage.

**Methods:**

The dependence of full field and half field pattern VEPs on retinal areas and cortical generators is reviewed and applied to the interpretation and evaluation of multichannel half field pattern VEPs.

**Results:**

There are predictable differences in the trans-occipital distributions of components of monocular full, and half field, pattern-reversal and full field, onset-offset VEPs. In combination, the differing distribution and dependence of these components on foveal and macular fields can help to identify and localise chiasmal and retro-chiasmal dysfunction and distinguish this from trans-occipital distribution due to individual variations of cortical architecture. A decision tree synthesising published evidence and current practice is suggested to guide interpretation of trans-occipital VEP distributions.

**Conclusion:**

The routine application of two additional lateral channels to acquire multichannel VEPs is quick, easy and adds clinical diagnostic value. The combination of full and half field pattern-reversal and fullfield, onset-offset VEPs can help evaluate chiasmal and retro-chiasmal visual pathway function, and minimise false positive interpretation of asymmetric VEP distributions, which may be due to cortical architecture or cranial anatomy alone.

## Introduction

Multi-channel VEPs (mcVEPs) are needed to evaluate chiasmal or retro-chiasmal function [1, 2]. Yet practical advice about how to interpret mcVEPs is sparse. The seminal study describing paradoxical lateralisation of half field pattern reversal mcVEPs was published in Nature in 1976 [3]. This paper sparked decades of debate [4]. Gradually, other brain imaging techniques validated Barrett et al.’s 1976 finding [5], and half field, pattern reversal mcVEPs have become accepted, and indeed advocated, for patients who cannot accurately complete visual field assessment, such as children [6]. Several studies have concurred that mcVEPs are achievable in children before accurate perimetry [6].

A review of clinical studies using half field pattern reversal VEPs (HF-prVEPs) highlighted their high sensitivity for detecting chiasmal visual field defects, (89%) and also homonymous hemianopia (92%), [7]. A study by Handley and Cowe et al. [8] (paper in review) identified visual dysfunction in 11/56 (20%) children without nystagmus or oculocutaneous signs of albinism which would have been missed if only a single midline VEP channel was used. This highlights the diagnostic value of using mcVEPs routinely.

The purpose of this technical note is to review the technical and physiological considerations needed to evaluate clinical full field (FF) and half field (HF) pattern mcVEPs in children, based upon dipole orientation of VEP generators. It will not describe analysis techniques used to identify the chiasmal misrouting associated with albinism at different ages.

## Methods

### *Electrode placement*

McVEPs are recorded from a trans-occipital array of electrodes spaced equally around the midline, Oz or Iz referred to a midfrontal reference, described in the ISCEV VEP Standard 2025 update [2]. The typical positions based on % measures of half head circumference are O1, O2, PO7 and PO8 [1, 2, 9]. Others use the Queen Square method and specify the lateral distance in centimetres such as 5 cm either side of Oz in adults [10]. This cm spacing is a similar location to the wider array PO7 and PO8 for the average adult head circumference of 56 cm. The wider electrode placement provides better definition of HF-prVEP components arising from paramacular regions [3, 11].

### *Stimulus parameters: check width and field size*

The paradoxical features of HF-prVEP trans-occipital distribution are specific for the ISCEV Standard large (60′) check width presented in a large 30 degree field, and may not be evident if a smaller check width or field size is used.

## VEP trans-occipital distribution

### *Full field prVEP trans-occipital distribution*

**Fig. 1:**
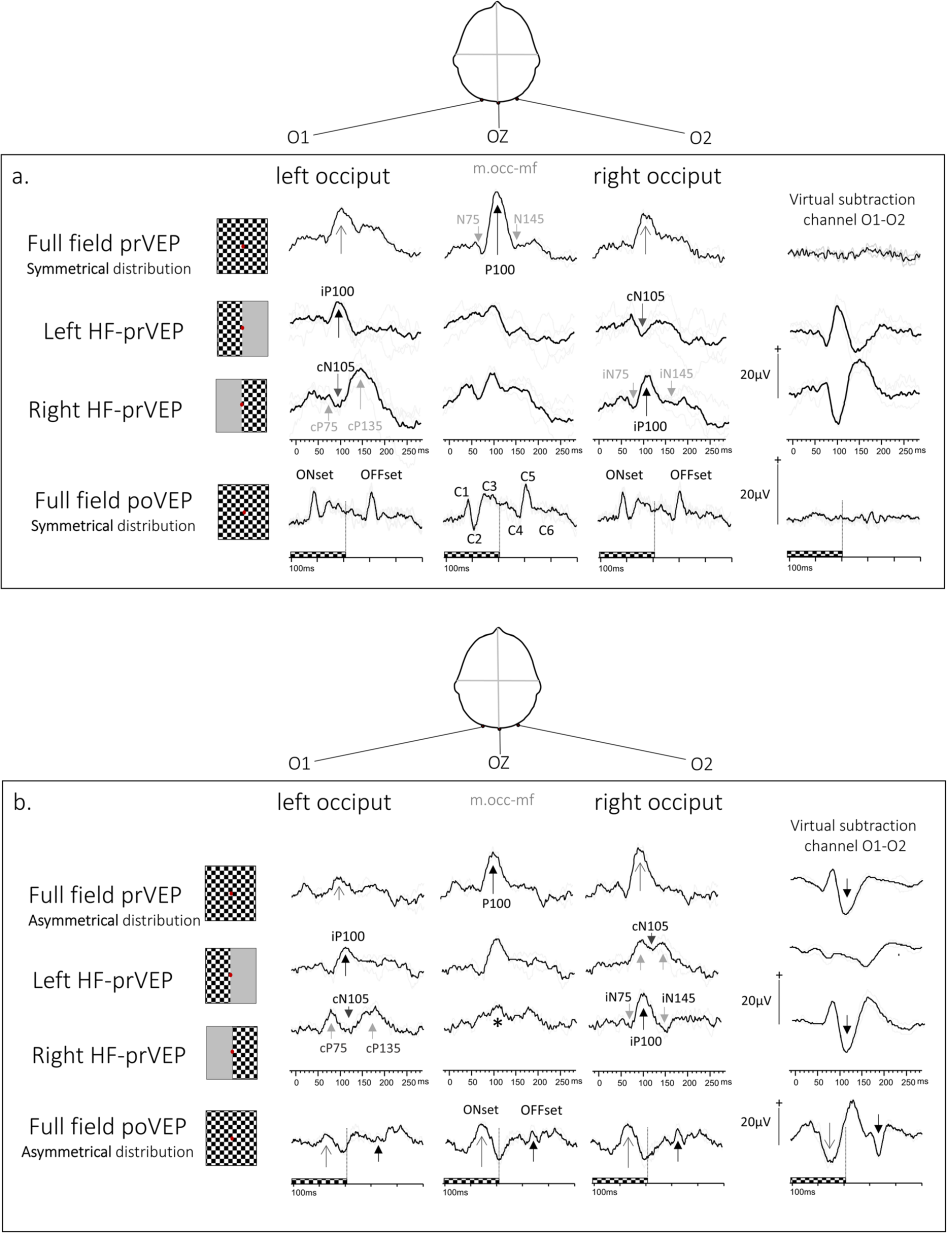

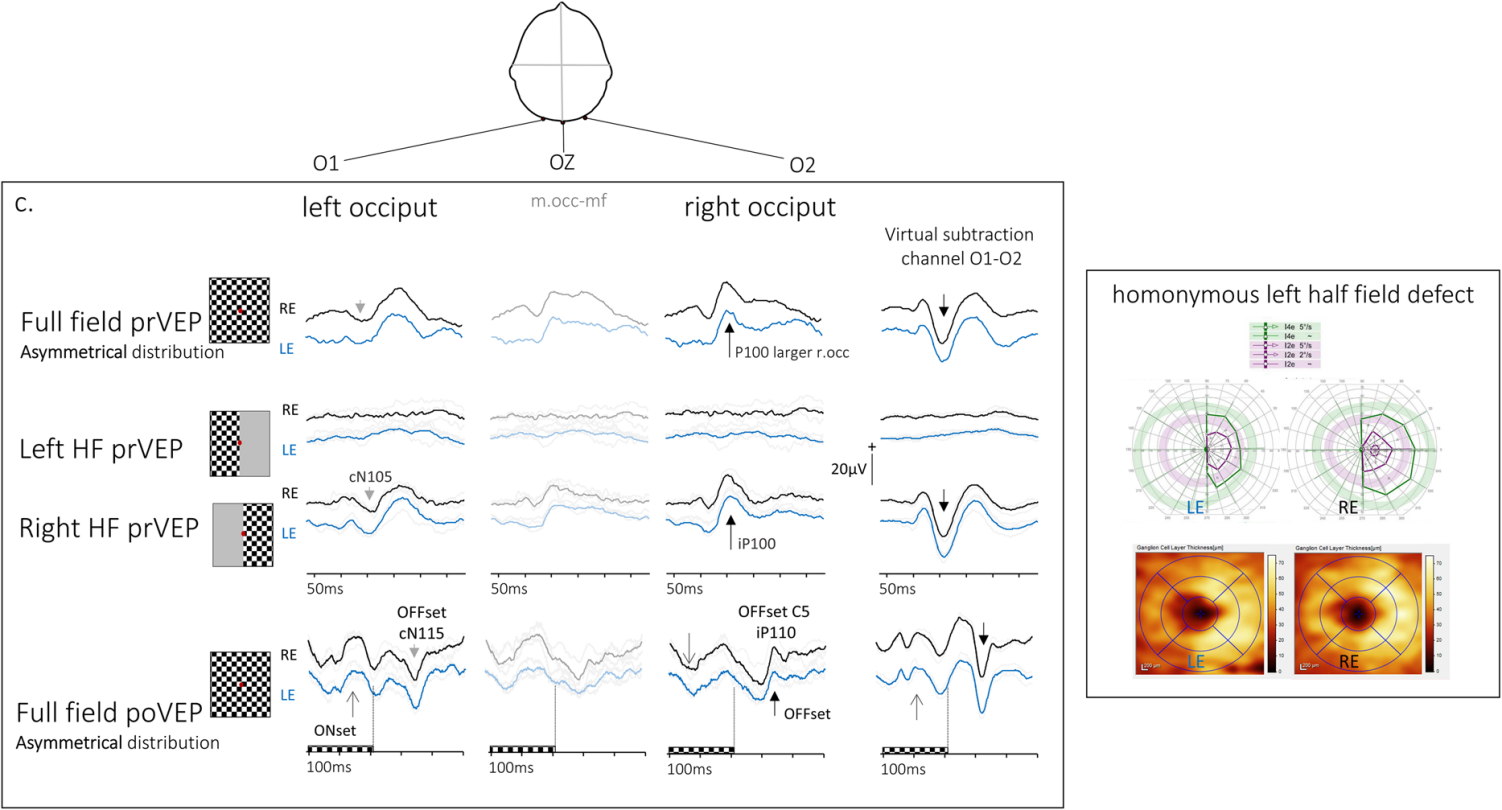
FF-prVEP trans-occipital distributions symmetric (**a**) and asymmetric (**b, c**), about Oz with constituent Left and Right HF-prVEP and nomenclature. The labelling of VEP peaks on the lateral channels uses the convention for naming a positive or negative peak, as P or N, followed by the peak time. The prefix i for ipsilateral and c for contra-lateral occiput are added with respect to the stimulated half field. **Fig. 1a** The top row shows a symmetrical trans-occipital distribution of a monocular FF-prVEP. The largest P100 amplitude is at the midline and smaller similar amplitudes on the right and left lateral channels (open arrows). The difference of O1–O2 shown in a virtual subtraction channel is a useful cross check of symmetry. As expected it is flat when the distribution is symmetrical about the midline. HF-prVEPs from the same eye are shown in rows below the FF-prVEP. HF-prVEPs peaks are lateralised. This case highlights the ‘paradoxical’ trans-occipital distribution of HF-prVEPs. The prVEP to the Left HF stimulation produces an iP100 ipsilateral to the stimulated field i.e. on the left occiput and a cN105 on the contralateral occiput. The Right HF-prVEP shows the opposite distribution, iP100 is seen on the right occiput. The difference of O1–O2 for the Left HF and Right HF-prVEPs are mirror images of each other. If summed they would cancel each other to produce a flat line—as seen for the FF-prVEP subtraction channel above. A FF-pattern Onset-Offset (po) VEP from an adult 55y is shown on the bottom row of Fig. 1a. This is also symmetrically distributed about the midline, as shown by the difference of lateral channels which is flat; ONset and OFFset VEPs are positive peaks on each occiput. **Fig. 1b** shows an asymmetrical trans-occipital distribution of a monocular FF-prVEP from a 5y child. The positive peak is larger over O2 compared to O1. The constituent Left HF and Right HF-prVEPs are shown below this. Each HF produces an appropriate iP100. This indicates that both right and left central half fields of this eye are functioning thus, the FF trans-occipital distribution is unlikely to be associated with a field defect of the central 30 degree field. In this example, the sum of the HF-prVEPs on each occiput explains the FF-prVEP distribution—the low amplitude over the left occiput is due to the cancellation of the Left HF iP100 and Right HF cN105. Interestingly, the Right HF-iP100 is well defined compared to the attenuated prVEP Oz P100 (marked with *) illustrating the advantage of mcVEP recording for HF stimulation. The bottom row shows the FF-poVEP. The ONset VEP morphology has the broad positive peak seen in children. The largest ONset VEP is over O2, the same as for the prVEP P100. The ONset VEP lateralises to the same occiput as the P100, suggesting that the skew in VEP distribution is due to individual retinotopic orientation of generators. In true pathology these peaks would lateralise to opposite occiputs (see Fig. 1c). **Fig. 1c** shows the distribution of FF- and HF-prVEPs and FF-poVEPs from each eye in a 15y patient with left homonymous hemianopia. The patient had a AV malformation that bled and awoke with a hemianopia. VA was preserved LogMAR −0.1 in each eye indicating some foveal sparing. The visual field plot and Spectralis OCT macular retinal ganglion cell (RGC) volume for each eye show the left half field defect for each eye with corresponding RGC volume loss. (N.B. Although the RGC volume corroborates the field loss in this specific case, it’s important to note that OCT findings do not always show this clearly in children with hemianopia). The top row of traces show that the FF-prVEP P100 is lateralised over the right occiput. This mimics the distribution of a functioning Right HF-prVEP, the cN105 seen on the left occiput. Specific stimulation of the left occiput. Specific stimulation of the Left occiput. Specific stimulation of the Left HF doesn’t elicit a response and confirms a profound Left HF defect affecting the 0–15 degree field of each eye. The FF-poVEP trans-occipital distribution shown on the bottom row shows how the lateralisation of ONset and OFFset VEPs can help if reliable half field stimulation is not possible. The OFFset VEP P110 behaves like the FF-prVEP P100 and is positive on O2, ipsilateral to the stimulated, functional field. An OFFset cN115 is seen on O1. The ONset VEP does not paradoxically lateralise. It is positive over the left occiput (from the left hemisphere), and negative over the right occiput. Effectively in this case of homonymous Left HF defect, the FF-poVEP behaves like a Right HF-poVEP. When ONset and OFFset VEP positive peaks lateralise on opposite sides of the head it is suspicious of a true field defect. Similarly if the ONset VEP and FF-prVEP positive peaks lateralise to opposite occiputs it is suspicious of a true field defect. The trans-occipital distribution of the VEPs produced by each stimulus were the same from each eye showing that there is a homonymous field defect

A prVEP to monocular 30 degree FF stimulation is the approximate algebraic sum of positive and negative components of HF-prVEPs generated by each hemisphere [12]. The largest P100 amplitude produced to FF pattern reversal stimulation is expected on the midline electrode Oz referenced to Fz, with smaller, similar amplitudes on each lateral electrode, providing a symmetrical voltage distribution about the midline (Fig. 1a top row).

An asymmetric FF-prVEP trans-occipital distribution (Fig. 1b top row) occurs when the amplitude on a lateral channel is larger than the midline or the opposite lateral channel. An abnormality of a FF-prVEP is regarded as an amplitude ratio of ≤ 1:3 from the two lateral channels 5 cm away from the midline [13, 14]. An asymmetric trans-occipital distribution of a monocular FF-prVEP is clinically informative by signposting a possible reduced contribution from either the nasal or temporal half field, and prompts additional investigation such as HF-prVEPs and/or FF-pattern ONset/OFFset VEPs (poVEPs) (Fig. 1a bottom row). It’s important to remember that a symmetrically distributed FF-prVEP sometimes can mask a half field defect [12, 15].

### *Half field prVEP trans-occipital distribution*

Hemianopic field deficits can be investigated specifically using HF pattern reversal stimulation that restricts activation to a specific hemisphere, irrespective of brain and skull architecture. Typically, large check (60′) patterns are presented in the central 0–15 degree nasal and temporal HFs. The resulting HF-prVEP interrogates hemisphere function and the sum of nasal and temporal HF-prVEPs can explain the FF-prVEP trans-occipital distribution to a centrally fixated 30 degree full field [15] (Fig. 1a, b).

#### Paradoxical lateralisation

Barrett et al. 1976 used the term paradoxical lateralisation to describe the trans-occipital distribution of HF-prVEPs. Paradoxical lateralisation is a characteristic of prVEPs produced by large check widths (60′) presented in a large stimulus field (30 degree) and a monopolar electrode montage using a mid-frontal electrode reference [1]. Paradoxical lateralisation is seen also for pattern OFFset VEPs but not for ONset VEPs [16]. The paradox is thought to arise due the orientation of the pattern reversal and offset generator neurons, which lie more obliquely within the posterior medial aspects of the calcarine sulcus (visual cortex) [3].

In contrast to the ISCEV Standard VEP monopolar montage, bipolar montages that are closer to the neural source and record near field responses, such as a voltage chain across the occiput or reference to the ipsilateral parietal region, will not paradoxically lateralise, but have variations. The clinical use of upper and lower half field pVEPs is less well established [7]. The lower visual field is mapped retinotopically to a gyrus closer to the occipital scalp electrodes. This feature is helpful for recording pattern VEPs if a child is reluctant to look centrally but will look at the top of a screen. There is more inter-individual variation in the sulcus representation of the upper field, but inter-ocular comparison of upper and lower field prVEP can be useful [7]. Multifocal VEPs can be valuable to monitoring visual fields an individual over time, though different electrode montages are used [17, 18].

### *Macular and paramacular visual field dependence of pattern VEP components*

Experimental simulation of visual field scotomata have helped to ascribe the dependence of the different pattern VEP waveforms on foveal, macular and paramacular retinal regions [10, 19]. These are summarised in Table 1. FF-pVEP peaks such as reversal N75, P100 and also ONset C2, C3 and OFFset C5 (P110) depend upon the macular field [2, 10, 20]. The main ipsilateral HF-prVEP iP100 depends upon foveal/ macular visual fields, whilst the main contralateral HF-prVEP cN105 depends upon paramacular regions. The paramacular HF-prVEP components cP75-cN105-cP135 can encroach into the midline channel of a FF-prVEP if a scotoma reduces the P100 contribution from the macular field. This can cause a bifid prVEP waveform with two positive peaks at the midline. A bifid waveform is seen clinically when optic atrophy affects the papillo-macular bundle or if there is a retinal maculopathy. HF stimulation can determine if a late or prolonged midline positive peak is a true P100 delay or is a HF paramacular peak cP135 [13].

**Table 1:** Summary of VEP waveform dependence on visual fields

VEP waveform dependence on visual field			
**FF prVEP**	**N75**	**P100**	**N145**
	*Macular*	*Macular*	*Macular*
**HF prVEP ipsilateral**		**iP100**	**iN145**
		*Foveal*	*Foveal*
**HF prVEP contralateral**		**cN105**	**cP135**
		*Para-macular*	*Para-macular*
**Onset VEP**	**C1 (P)**	**C2 (N)**	**C3 (P)**
	*Para-macular*	*Macular*	*Macular*
**OFFset VEP**	**C4 (N85)**	**C5 (P110)**	**C6 (N165)**
	*Macular*	*Macular*	*Macular*

The table summarizes the visual field dependence of VEP components produced to different stimulation. These were derived from component reduction with scotoma of different radii defined as foveal 0°–1.5°, macular 0°–8° and paramacular 6°–8°. All macular and foveal dependent VEP components become more prominent with the ISCEV Standard small (15′) check width. The large 60′ check is preferred for HF-prVEPs from 0 to 15 degree fields because it provides information about paramacular field components. Paramacular components are typically seen on the occiput contra-lateral to the stimulated field. Paramacular contra-lateral HF-prVEP components can show more inter-individual variability than ipsilateral HF-prVEP components and are evident more consistently with wider electrode spacing.

### *Uncrossed and crossed asymmetry of trans-occipital distributions*

The Queen Square group introduced the terms ‘crossed’ and ‘uncrossed’ asymmetries to describe monocular VEP distributions [3]. An uncrossed asymmetry is typical of homonymous hemianopia due to a lesion posterior to the chiasm affecting the pathway to one hemisphere. In this instance the FF-prVEP of each eye will resemble the HF-prVEP from the functioning hemisphere. Irrespective of which eye is stimulated the functioning half field produces an iP100 lateralised over the same occiput—an uncrossed asymmetry. (Fig. 1c.) The HF-prVEPs can confirm an abnormal response from the dysfunctional hemisphere. A crossed asymmetry is typical of bitemporal or binasal hemianopia, or anatomical chiasmal disproportion such as albinism or achiasmia [14]. The ip100 again follows the strongest functional field, but in this case the occipital distribution will change laterality or ‘cross over’ as the stimulated eye changes.

## Multi-channel VEP evaluation

The discrimination of each HF-prVEP and its contribution to the FF-prVEP is achieved by analysing iP100 amplitudes and peak times from lateral channels (Fig. 1), and by considering the prominence of cN105 (Fig. 1a). The reference limit for the ratio of HF-prVEP iP100 amplitude from an eye is regarded as ≤ 1:2 (from lateral electrodes 5 cm from midline in adults) [13, 14]. The variation of this reference range highlights the need for corroborating evidence rather than reliance on absolute measures. Any abnormalities should be reproducible and ideally evident on more than one measurement [21]. There is usually no marked difference between iP100 from the nasal and the temporal fields of each eye [14]. Abnormal HF-prVEP peak times may be prolonged, associated with cP135, but also atypically early associated with predominance of cP75. Published reference data for the lateral channel VEP amplitude and peak time changes during maturation are lacking and require further study.

### *Decision trees for multi-channel VEPs*

**Fig. 2:**
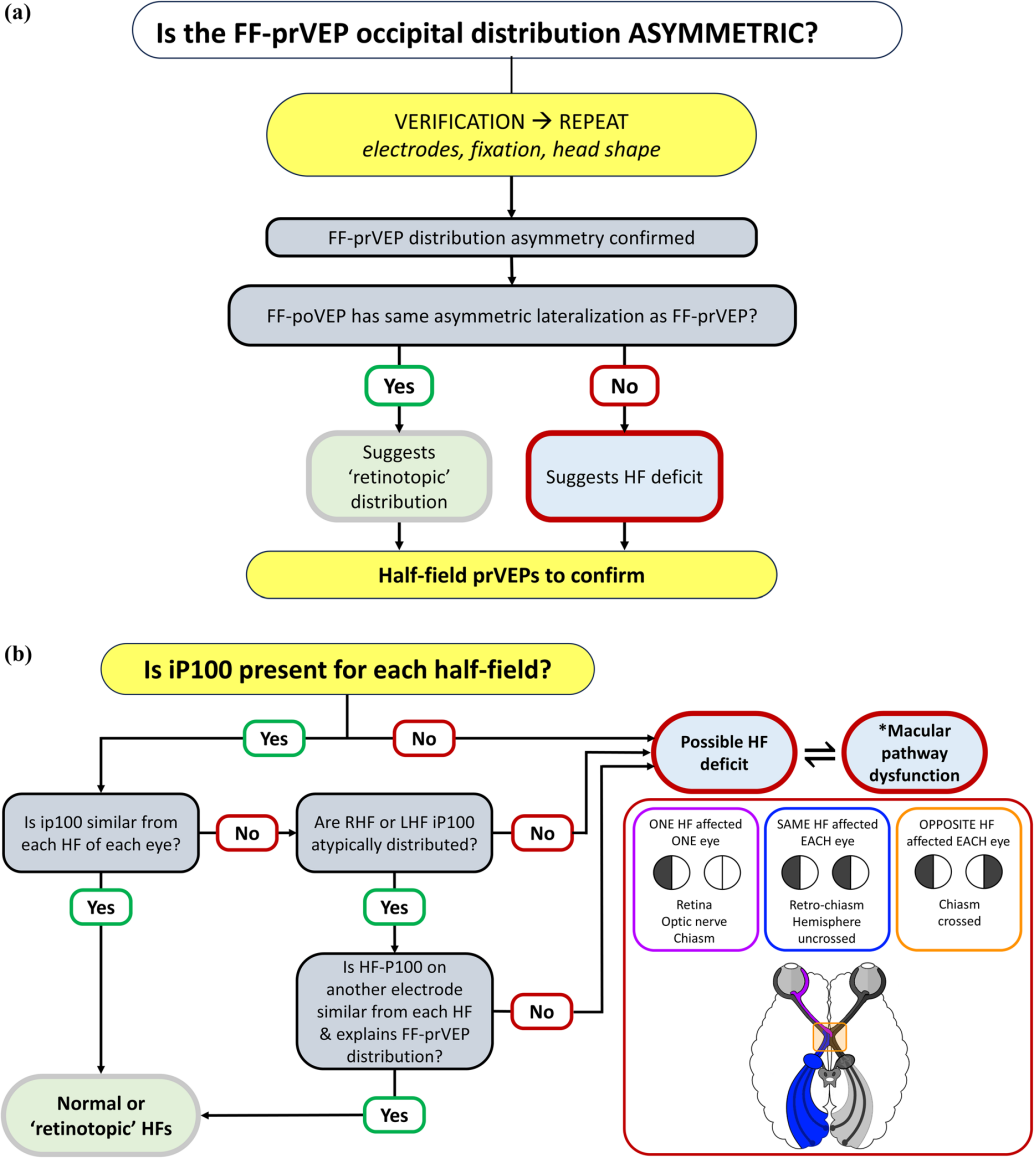
Decision trees for multichannel VEPs. **a** Full field pattern reversal VEPs (FF-prVEP) decision tree. The first task when the FF-prVEP has an asymmetric trans-occipital distribution about the midline is verification. Check if the patient has a head tilt, and/or non- central fixation and/or an asymmetric head shape. Next, verify the midline electrode placement and that the lateral electrodes are equally spaced around the midline with no vertical displacement. This can be hampered by skull asymmetry due to postural plagiocephaly or craniosynostosis or VP shunt. These should be noted along with any adjustments before repeating the test. If the asymmetric FF-prVEP is confirmed, record a FF-poVEP. If the poVEP distribution has the same asymmetric lateralisation as FF-prVEP the cause is most likely due to individual physiological variation in cortical generators termed here a ‘retinotopic’ distribution. Confirmation with HF-prVEP is still desirable where age and/or ability permits. If, however, the FF-poVEP and FF-prVEP have opposite asymmetric distributions, i.e. the largest peak lateralises on opposite occiputs, it is suspicious and needs further investigation (Fig. 1c). **b** Half field pattern reversal VEPs (HF-prVEP) decision tree. If the HF-prVEP produces an iP100 it shows that this HF is responsive. If the iP100 amplitudes and peak time are similar from the same HF of each eye and the sum of nasal and temporal HF-prVEP lateral channels explains the FF-prVEP distribution asymmetry a real visual field defect affecting the central 0–15 degrees is unlikely (follow the Yes pathway). If the iP100 is small or not evident (follow the No path), it can indicate a HF defect or a central scotoma reducing iP100 such that the cN105 encroaches onto the midline giving a bifid waveform (*). Such a central scotoma may be caused by optic atrophy or maculopathy. In each of these scenarios HF-prVEP amplitudes and peak times from each eye and each half field should be compared. If iP100s are dissimilar but atypically distributed, it’s helpful to inspect the multichannel array to see if iP100 on a different electrode is larger and more comparable from each eye, and to iP100 from the opposite HF. If this is the case then a ‘retinotopic’ distribution is likely. If however iP100s are dissimilar but typically distributed it is suspicious of a HF defect (follow the No pathways). The schematic on the right shows the locations of pre-chiasmal, chiasmal and retro-chiasmal lesions and the HF defects they may cause; a left eye, left HF defect (magenta), an uncrossed retro-chiasmal homonymous hemianopia with small amplitude iP100 from the same HF of each eye (blue) and a crossed asymmetry indicating chiasmal dysfunction with bitemporal (or binasal) field defects. Pathology can affect one eye more than another. HF-prVEPs may not be identically affected but may show similar trends in distribution

The flow charts provide a recording and evaluation guide to help distinguish VEP trans-occipital distributions associated with retinotopic individual variations in healthy cortical VEP generators from those due to visual pathway pathology. In all cases a HF-prVEP is the desirable gold standard, but not always possible. For children unable to attend the edge of a HF stimulus reliably, the difference between paradoxical lateralisation of mc FF-prVEP and faithful lateralisation of FF-poVEPs can be clinically helpful. As mentioned any abnormalities should be internally consistent, and interpreted cautiously in the clinical context of other findings [21] (Fig. 2).

## Conclusion

This technical note describes an approach for evaluating mcVEPs in routine practice. Additional lateral channels are quick to apply and add clinical diagnostic value. In combination, the complementary evidence from FF-prVEPs and HF-prVEPs, that show paradoxical lateralisation, and FF-poVEPs that faithfully represent the activated hemisphere, can help to distinguish chiasmal and retro-chiasmal visual pathway function and minimise false positive interpretation of asymmetric VEP distributions, which may be due to cortical architecture or cranial anatomy alone.

## Abbreviations

mcVEP: Multichannel VEP
prVEP: Pattern reversal VEPs
poVEP: Pattern onset/offset VEPs
ONset VEP: Pattern onset VEPs
OFFset VEP: Pattern offset VEPs
FF: 30 Degree square full field
HF: 0–15 Degree nasal or temporal half field

## Declarations

### Statement on the welfare of animals

This research did not involve animals.
